# Glycemic Response to Black Beans and Chickpeas as Part of a Rice Meal: A Randomized Cross-Over Trial

**DOI:** 10.3390/nu9101095

**Published:** 2017-10-04

**Authors:** Donna M. Winham, Andrea M. Hutchins, Sharon V. Thompson

**Affiliations:** 1 Department of Food Science & Human Nutrition, Iowa State University, Ames, IA 50011, USA; 2 Department of Health Sciences, University of Colorado Colorado Springs, Colorado Springs, CO 80918, USA; Andrea.Hutchins@uccs.edu; 3 Division of Nutritional Sciences, University of Illinois at Urbana Champaign, Urbana, IL 61801, USA; svthomp2@illinois.edu

**Keywords:** legumes, pulses, beans, glycemic response, blood glucose, post-prandial, insulin, insulin response

## Abstract

Legumes, such as black beans (*Phaseolus vulgaris* L.) and chickpeas (*Cicer arietinum* L.), have a low glycemic index, and may reduce the glycemic load of meals in which they are included. Although the low glycemic response of beans consumed alone has been documented, few studies have examined the glycemic response to traditional food combinations such as black beans and rice or chickpeas and rice. This randomized cross-over study examined the glycemic and insulinemic impact of 50 grams of available carbohydrate from three test meals: plain white rice (control), black beans with rice, and chickpeas with rice among healthy adult women (*n* = 12, 18–65 years). Treatments were consumed on different mornings, a minimum of 7 days apart. Blood samples were collected at time 0 (fasting), and at 30, 60, 90, and 120 min postprandial, and were subsequently analyzed for glucose and insulin concentrations. Glucose response based on the incremental area under the curve showed a significant difference by treatment (*p* = 0.027). Changes in blood glucose concentrations were significantly different for the black bean meal and the chickpea meal in comparison to rice alone at 60 min (*p* = 0.026 and *p* = 0.024), 90 min (*p* = 0.001 and *p* = 0.012) and 120 min post prandial (*p* = 0.024; black bean meal). Findings indicate that combinations of black beans and chickpeas with white rice improve glycemic response, providing evidence that has promising implications for dietary guidance to reduce postprandial glucose and related health risks through traditional food patterns.

## 1. Introduction

Legumes and other pulses (dried peas, lentils) have been a staple food for millennia in a majority of cultures, and often define a region’s cuisine [1]. Two of the most frequently consumed dry grain pulses are black beans (*Phaseolus vulgaris* L.) and chickpeas or garbanzo beans (*Cicer arietinum* L.). Black beans and chickpeas are commonly consumed in many regions around the world, including India, Latin America, and the Middle East [1]. These legumes, and others, are frequently eaten as part of a meal with high carbohydrate foods such as rice, tortillas, or potatoes. Black beans and chickpeas with rice are part of classic cultural dishes like *feijoada* in Brazil and *chole biryani* in India [1].

Legume consumption is connected to a vast number of health benefits, including increased satiety, lengthened longevity, improved body weight, and the prevention and treatment of chronic conditions like metabolic syndrome, type 2 diabetes, and coronary heart disease (CHD) [2,3,4,5,6,7,8]. Legumes are high fiber, low glycemic foods that contain a considerable amount of soluble fiber and resistant starch, and a higher ratio of slowly digestible to readily digestible starch than other carbohydrate foods [9]. Soluble fiber contributes to an increase in self-reported satiety and a reduced rate of both gastric emptying and nutrient access by alimentary digestive enzymes. Legume-derived resistant starch and slowly digestible starch are also associated with improved glycemic response and lower postprandial glucose concentrations, which can improve glycemic control among individuals with insulin resistance and type 2 diabetes [10,11]. Legumes are also a source of plant protein and anti-nutrient factors such as polyphenols and phytonutrients. As with other dietary sources of protein, legumes promote the release of satiety hormones such as cholecystokinin and glucagon-like protein 1, hormones that may be responsible for the 31% increase in self-reported satiety observed when pulses were compared to control treatments [12].

Although legumes remain important and essential foods, shifts to a more Western diet pattern are occurring in many countries, regardless of migration [13]. Urbanization and easier access to inexpensive processed foods have altered the nutrition environment, even in rural areas [13,14]. For immigrants who move to a developed country, dietary acculturation may become a necessity due to the lack of familiar foods. Regardless of the source of dietary change—migration or nutrition transition—the shift to a Westernized diet is linked to an increased risk of diabetes, cardiovascular disease, some types of cancers, and related conditions such as metabolic syndrome and obesity [14]. The development of type 2 diabetes has clear connections with diet and lifestyle [13]. Since the 1980s, global estimates of diabetes indicate that rates have increased by 60% among women, and by two-fold among men [15]. Dietary patterns that promote elevated postprandial glucose excursions can damage tissues, reduce vascular endothelial function, and, over time, lead to degeneration of normal physiological functioning of the pancreas. Controlling or limiting elevated postprandial glucose is beneficial for non-diabetic individuals as well as those who have advanced to a diseased state [16].

The fiscal impact of these conditions, which are so intertwined with diet and lifestyle, is of great concern. US healthcare and economic costs connected with type 2 diabetes treatment alone were estimated to be $245 billion in 2012, up from $174 billion in 2007 [17]. Estimates for CHD costs on a global level may reach as high as $20 trillion over the next two decades [18]. Despite educational campaigns and pharmaceutical advances with regard to the treatment of conditions characterized by aberrant blood glucose and insulin homeostasis through diet and medications, the incidence of type 2 diabetes and CHD continues to rise, and remain among the top ten causes of death in the US and internationally [15,19].

Retention or promotion of traditional foods with nutritional benefits such as legumes, is a logical strategy to improve diets in the face of changing food environments and promote healthier eating patterns [13,20]. Legumes have superior nutrient profiles in comparison to other common dietary staples, such as rice and corn [21]. For example, black beans and chickpeas contain more than 7 g of protein, while long-grain white rice and kernel corn contain only 2 g per 1/2 cup serving of cooked food. Folate concentrations are also higher in legumes (black beans 128 μg; chickpeas 141 μg; rice 2 μg; corn 17 μg), as is iron (black beans 1.81 mg; chickpeas 2.37 mg; rice 0.16 mg; corn 0.34 mg) [22].

Given the substantial evidence for legume-related health benefits, the US Dietary Guidelines for Americans (DGA) first recommended their inclusion in the 2005 DGA [23]. The 2015 DGA recommends 1 to 1.5 cups of legumes be consumed per week for a 2000 kcal diet. The DGA includes these plant foods among the vegetable and protein food categories of MyPlate [23]. Despite this guidance, most Americans fail to reach the DGA legume recommendation [24]. The current US Western diet pattern often lacks a legume-based staple food, instead featuring meat as a primary protein source [1]. Globally, annual per capita consumption patterns range from 34 kg in Burundi, 18 kg in Nicaragua, 11 kg in India, to only 4 kg in the United States (US) [21]. US per capita consumption masks the fact that legume consumption is highest among ethnic groups and minorities [24]. Legume intakes may decrease as individuals acculturate to a Western diet in the US [25,26], or internationally as individuals experience nutrition transition changes in their country of origin [27]. In the US, rice consumption is about 9.5 kg per capita. Like legumes, rice is consumed in greater quantities among multicultural individuals [28]. While some Asian countries like Cambodia and Vietnam report rice consumption per capita of over 110 kg, it is also high in the Caribbean Region at 70 kg, and in South America at 45 kg [29]. In most of these settings the rice is paired with a legume [1].

Although the low glycemic response of beans alone has been documented [30,31], evidence for the metabolic impact of traditional food combinations such as black beans and rice, or chickpeas and rice has been limited and, among existing trials, results have been mixed [4,32,33,34,35,36,37,38,39,40]. Investigating the glycemic and insulinemic impact of black bean and chickpea consumption in a traditional meal setting can support legume retention in traditional diets and encourage dietary recommendations by health providers to increase consumption of these culturally valuable plant foods. As risk of metabolic syndrome and type 2 diabetes increases among ethnic minorities and the overall US population, effective high-fiber, high-protein plant foods like legumes may lower disease burden and promote health among ethnic minorities and the overall US population [20].

Accordingly, the objective of this study was to examine the glycemic and insulinemic response to two dry bean varieties (black beans (*Phaseolus vulgaris* L.) and chickpeas (*Cicer arietinum* L.)), in combination with white rice as a culturally appropriate complementary food, in comparison to a white rice control meal among adult women. We hypothesized that the inclusion of whole black beans or chickpeas with white rice would reduce the glucose and insulin response when compared to the white rice control.

## 2. Materials and Methods

### 2.1. Study Population

Adult women, aged 18–65 years, were recruited from the greater metropolitan Phoenix area to participate in the 3 × 3 randomized cross-over trial. Exclusion criteria included physician diagnosis of type 1 or type 2 diabetes, behaviors or health conditions known to influence glucose or insulin concentrations (e.g., smoking, gastrointestinal conditions, BMI ≤ 19 or ≥ 35 kg/m^2^, weight changes ±5 kg within 6 months, current pregnancy or breastfeeding), or allergy to beans or latex. Participants were also excluded if they were currently taking medications known to affect glucose or insulin concentrations. Habitual consumption of medications not known to impact glucose or insulin metabolism was permitted if the participant was on the current treatment dosage for >6 months and dosage was not altered during the study period. The study protocol was approved by the Bioscience Committee of the Institutional Review Board at Arizona State University (Human Subjects Protocol Number 0712002492) and all participants provided written, informed consent. Twenty-one individuals (19 women, 4 men) were screened for the study. Due to the potential for sex-related confounding in metabolism and body size, and insufficient enrollment of men for adequate statistical comparison, male participants were excluded from the study. Of the 19 eligible women, 6 declined to participate and 13 were enrolled in the study. One participant completed one test day, but declined to continue the study for personal reasons. Three women completed two test days and 9 women completed all three test days.

### 2.2. Study Design

This randomized cross-over study included three treatments: (1) white rice (control); (2) black beans and white rice; and (3) chickpeas and white rice. Test meals were consumed on different mornings at least one week apart. Treatments were portioned by gram weight and contained equal available carbohydrate content of 50 g. Black beans and chickpeas have similar carbohydrate content per gram weight despite being different legume species [22].

During the three days prior to testing, participants were asked to consume one white, plain bagel (56 g carbohydrate) each day to ensure adequate carbohydrate consumption minimize influence of glycogen depletion on postprandial glucose. Participants selected a pre-testing evening meal consisting of a commercially produced submarine sandwich, potato chips, and cookie consumed with water. Participants were required to consume the same evening meal the night before each test day. Evening meal standardization was conducted to avoid confounding from the second meal effect [41]. After consuming the provided meal on the eve of testing, participants were required to fast and to drink only plain water until they arrived at the study location 12 h later. Participants were also asked to refrain from alcohol consumption and light, moderate, or heavy activity for 24 h prior to testing.

Upon arrival at the test site, participants were confirmed to be fasting and compliant with study procedures. Participants were weighed in light clothing, without shoes, using a digital scale (Seca Model 880; SECA, Hamburg, Germany). Height was assessed using a wall-mounted stadiometer (SECA, Chino, CA, USA) on the first test day meeting. After fasting blood sample collection, participants consumed one of the three test meal options within 5–10 min under researcher supervision. Meal consumption duration was not significantly different between treatments.

Whole blood samples were collected at 30, 60, 90, and 120 min post-treatment by a trained phlebotomist for determination of glucose and insulin concentrations. Plasma glucose concentrations were assessed using the colorimetric glucose oxidase method (Sigma Diagnostics, St. Louis, MO, USA). Insulin concentrations in serum were determined utilizing the Immulite 1000 (Diagnostic Products Corporation, Los Angeles, CA, USA). Results for glucose and insulin are presented as net change from fasting values. Fasting glucose concentrations were not significantly different between treatment test days and values confirmed that participants were fasting. A portion of the first blood sample on the first study test date was sent to an outside lab (Sonora Quest, Tempe, AZ, USA) for analysis of hemoglobin, triglyceride, total cholesterol, low-density lipoprotein (LDL), high-density lipoprotein (HDL), and very-low-density lipoprotein (VLDL). Hemoglobin was assessed to evaluate the presence or absence of iron deficiency anemia and lipid profiles were evaluated to provide information on risk factors for metabolic syndrome and heart disease.

### 2.3. Test Meals

Participants received the three test meals in random order. Bean treatment meals were composed of a 1/2 cup of plain black beans or chickpeas (Bush Brothers & Company, Knoxville, TN, USA) and 15 g of brine from the canned beans (added for flavor) along with 1/2 cup of plain steamed long grain white rice (Great Value, Bentonville, AR, USA). The control meal contained 3/4 cup of the same white rice. The mean glycemic index (GI) value of long grain rice was found to be 80 ± 3 across ten studies and it is considered to be a high-GI food [31,42]. Nutrient composition of test meals is shown in Table 1. Each meal provided 50 g of available carbohydrate, which was calculated as the difference between the dietary fiber and total carbohydrate values presented on the manufacturer’s nutrition facts label [43,44,45]. Fifty grams of carbohydrate is a standard quantity used to test glucose response among persons with and without type 2 diabetes [46,47,48]. White rice was prepared in an electric automatic rice cooker based on the manufacturer’s instructions (RC400; Black & Decker, Miami Lakes, FL, USA). Dry rice weight and water volume were standardized to gram weights for preparation consistency. Proportions of 945 g of bottled drinking water was added to 420 g of dry white rice and steamed for ~30 min in the rice cooker. The canned beans were drained, but not rinsed, and heated in a microwave for 1 min at medium power. The test meal was prepared by weighing out the cooked rice, then adding the appropriate weight of warmed beans, and 15 g of the drained can liquid for moisture. A digital food scale was used for gram weight determination, and was tared after the addition of each food item (Salter, Fairmont, MN, USA).

### 2.4. Data and Statistical Analysis

**Figure 1:**
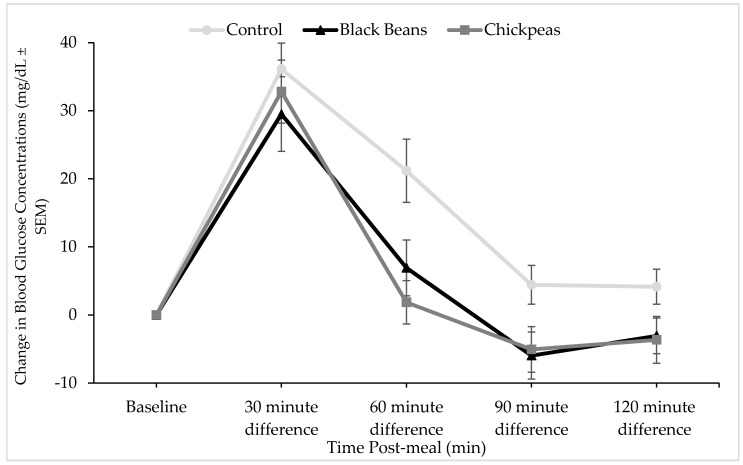
Effect of bean interventions on change in plasma glucose concentrations from fasting. Points denote mean glucose concentration at each data collection timepoint and error bars denote standard error of means. Mean fasting glucose values were not significantly different between treatment groups (91.68 (SEM 2.42), 89.54 (SEM 1.83), and 89.64 (SEM 5.70) mg/dL for white rice, black beans and white rice, and chickpeas and white rice, respectively). Results of a post-hoc paired t-test indicated that the glucose response curve was significantly different at 60 (*p* = 0.026), 90 (*p* = 0.001), and 120 (*p* = 0.024) for black beans (*n* = 12), and at 60 (*p* = 0.024) and 90 (*p* = 0.012) for chickpeas (*n* = 9) compared to control (rice alone). A trend was observed at the 120 min timepoint between chickpeas and rice and the white rice control meal (*p* = 0.072).

**Figure 2:**
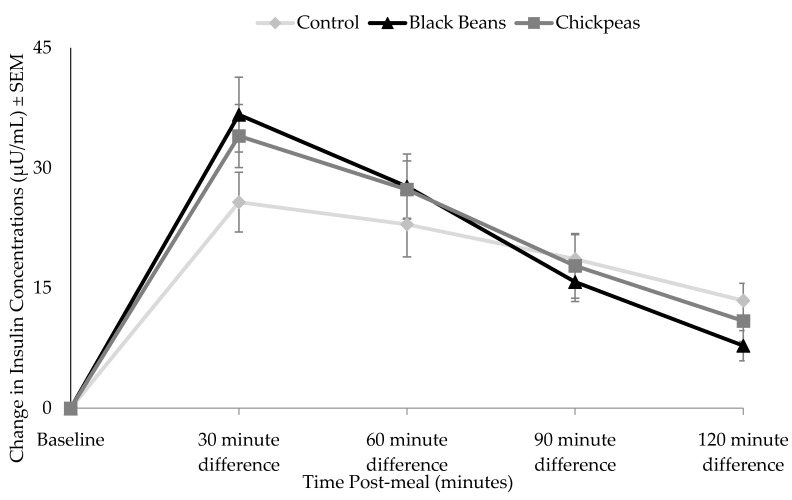
Effect of bean treatments on change in plasma insulin from fasting values. Points denote mean insulin concentration at each data collection timepoint and error bars denote standard error of means. Results of a post-hoc paired t-test indicated that the insulin response curve was significantly different at 30 (*p* = 0.037) for black beans (*n* = 12), and at 30 (*p* = 0.026) for chickpeas (*n* = 9) compared to the white rice control meal.

**Figure 3:**
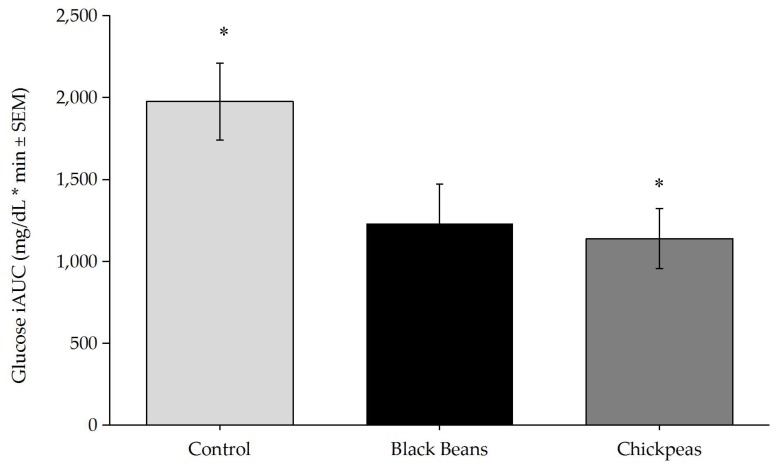
One-way Analysis of Variance of glucose iAUC was significantly different by treatment (*p* = 0.027). Tukey post-hoc tests indicated a significant difference between chickpeas and control (*p* = 0.047), and a trend was observed between black beans and control (*p* = 0.058). * *p* < 0.05. Bars denote mean insulin iAUC values and error bars denote standard error of means.

**Figure 4:**
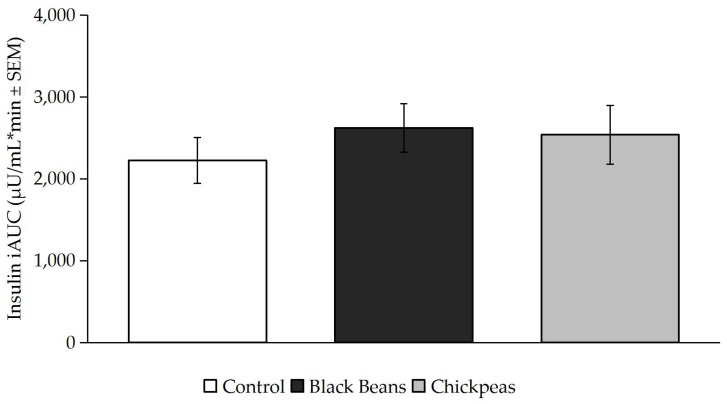
Insulin iAUC was not significantly different by treatment (*p* > 0.05). Bars denote mean insulin iAUC values and error bars denote standard error of means.

Timepoint differences between fasting and post-treatment glucose and insulin concentrations were determined (Figure 1 and Figure 2) and incremental area under the curve (iAUC) calculations were completed using the trapezoidal rule (Figure 3 and Figure 4) [49]. The iAUC for blood glucose and insulin were assessed between 0–60 and 0–120 min postprandial for all participants. Multivariate analysis of variance (MANOVA) for repeated measures with time and diet as factors was used to evaluate differences in glucose and insulin measures between the three meal treatments. Following a significant MANOVA, paired *t* tests were used to identify differences between specific bean treatments and the rice control. All continuous variable data are reported as mean ± standard error. SPSS Statistics software version 24.0 (IBM Corporation, Somers, NY, USA) was used for statistical analyses. *A priori* power analysis at 80% power with an effect size of 0.5 for MANOVA repeated measures between factors indicated that a sample size of 12 individuals were required [50]. The level of significance was *p* ≤ 0.05.

## 3. Results

### 3.1. Participant Characteristics

Descriptive statistics for the 12 participants at study entry are shown in Table 2. All participants self-identified as Caucasian and two identified as having Hispanic ethnicity. Body weight and body mass index (BMI) did not significantly differ between test days (data not shown). Mean BMI was within the normal range. Two participants were classified as overweight. Mean lipid concentrations were in the optimal ranges [51]. One participant displayed an elevated total cholesterol concentration of 267 mg/dL. The participant’s HDL cholesterol was also high, indicating a total cholesterol/HDL ratio of 4.0, which was considered acceptable by recommendations at the time of data collection [49]. None of the women were classified as anemic (hemoglobin <12 g/dL) [52].

### 3.2. Glucose and Insulin Responses

Timepoint differences in glucose concentrations were significantly lower at 60 and 90 min postprandial for black beans and rice (*p* = 0.026 and *p* = 0.001, respectively), and chickpeas and rice (*p* = 0.024 and *p* = 0.012, respectively) as compared with the white rice control meal. A significant reduction in postprandial glucose concentrations was also observed for the black bean and rice meal at the 120 min timepoint (*p* = 0.024). The glucose response to chickpeas and rice trended lower (*p* = 0.072) than the control meal at 120 min postprandial (Figure 1). Blood glucose iAUC values between 0 and 120 min were significantly different between treatments at the main effect level (*p* = 0.027; Figure 2). Tukey post hoc tests indicated a significant difference between the rice-only control and the chickpeas and rice meal (*p* = 0.047) and a trending difference between the rice-only control and the black beans and rice meal (*p* = 0.058)

Timepoint differences in insulin concentrations were significantly higher at 30 min postprandial for the black beans and rice (*p* = 0.037) and chickpeas and rice (*p* = 0.026) than for the white rice control meal (Figure 3). Blood insulin iAUC values assessed between 0 and 120 min postprandial were not significantly different between treatments (*p* > 0.05; Figure 4).

## 4. Discussion

Findings of this randomized, cross-over trial indicate that a half cup of whole black beans and chickpeas in combination with white rice reduced glycemic response among adult women without diagnosed diabetes compared to the rice-only control. The ability of black beans and chickpeas to mitigate the high GI of rice is consistent with many, but not all, studies that have explored the impact of whole dry beans on postprandial glucose and insulin when consumed with a high GI food among healthy subjects and individuals with insulin resistance and type 2 diabetes [4,32,33,34,35,36,37,38,39,40].

Black bean and rice and chickpea and rice meals decreased timepoint differences in glucose concentrations at 60 and 90 min postprandial compared to white rice control, but only black beans demonstrated a lower timepoint difference in glucose concentration at 120 min (Figure 1). Despite standardization based on 50 g of available carbohydrate, the black beans and rice meal contained slightly more fiber and protein compared to the chickpeas and rice meal. This difference, albeit small, may have extended the glucose lowering impact of black beans to the 120-min timepoint. In a project of similar study design, we observed that black, pinto, and dark red kidney beans showed significant decreases in postprandial glucose in comparison to a rice control among adults with type 2 diabetes [36].

The insulin incremental area under the curve (iAUC) did not significantly differ between treatments (Figure 4), but was slightly higher for the two bean and rice treatments than for rice alone. These increased values may have been driven by the difference in insulin concentrations for the black beans and rice and chickpeas and rice at the 30 min timepoint (Figure 3). As no other significant differences in insulin concentrations were observed at other timepoints, not only did the impact of the black beans and chickpeas on insulin differ from their effect on glucose, the increase in insulin they elicited at 30 min offers a possible explanation for the decreased glucose concentrations at 60 and 90 min for both interventions. It is also reasonable to postulate that the early increase in insulin served to move glucose out of the bloodstream and into the cells more rapidly after consumption of black beans and rice or chickpeas and rice versus white rice alone. Mixed responses in insulin AUC were observed among male and female participants with overweight and obesity following an 8-week intervention involving five cups of yellow peas, chickpeas, navy beans, and lentils per week in comparison to dietary counselling for energy restriction [51]. Like the present study, a significant glucose AUC reduction was observed among all participants with the pulse treatment (20.1% vs. 5.6%), but insulin AUC was reduced by 13.9% among female participants and increased by 27.3% among men [53].

Black beans originated in South America, and are most frequently eaten in Latin American and Caribbean cuisine. Chickpeas or garbanzo beans were originally cultivated in Europe, and are most commonly consumed in the context of Middle Eastern and Indian dishes [1]. Retention of legume consumption within traditional diets improves short term glycemia and insulinemia, and reduces the risk of chronic conditions such as CHD and type 2 diabetes, evidence which encouraged the current DGA recommendations in the US [23]. Importantly, our findings indicate that even a 1/2 cup of beans can produce reductions in postprandial glycemia. Legumes contain a variety of essential nutrients, and fall within multiple food group categories. Increased bean consumption improves dietary fiber and plant protein consumption—two dietary components that are lacking in US diets—and lower chronic disease risk [54]. As chronic disease incidence increases alongside the rising prevalence of obesity [14], determining culturally relevant dietary factors that lower disease risk is of high importance. Consumption of food combinations like black beans and rice, or chickpeas and rice, historically important components of traditional diets, often declines with acculturation to a Western diet [20] and corresponds to an increase in obesity and type 2 diabetes [13].

Strengths of the present study include reductions in confounding by study procedures and the use of translatable quantities of bean treatments. As this study was conducted among healthy individuals our study results may not have been impacted by chronic disease-related confounders. The consumption of the same pre-treatment evening meal and the minimum one-week separation between test days may have also lowered differences in glucose and insulin response due to the second meal effect. A half cup of beans was provided to study participants during treatment days. This quantity is likely more representative of actual per meal consumption and, given our results, indicates that even modest increases in bean consumption can produce beneficial effects in an acute setting. The translation of a bean and rice meal is an additional strength, as beans are rarely consumed in isolation [1].

The limitations of the present study should be noted. This was an acute study which cannot provide evidence of the long-term effects of bean intake. We relied on self-reported health status during screening and assessed blood lipids and other biomarkers at the first testing visit. Thus, while participants were considered healthy for the purposes of the present study, it is possible that unknown or undisclosed health conditions impacted our findings. We also did not control for stage of menstrual cycle during data collection. Some studies have suggested that minimal increases in blood glucose may occur during the follicular and luteal phases of the menstrual cycle among normoglycemic women [55,56]. As the present study used a cross-over design, and net glucose and insulin changes were measured over each treatment day, we believe any potential differences due to menstrual cycle were minimized by the methodology. However, it is possible that the observed differences could be influenced by hormonal changes. Additionally, our findings cannot be generalized to men or to individuals with chronic conditions such as type 2 diabetes, although we have previously observed acute reductions in postprandial glycemia with bean and rice treatments among individuals with type 2 diabetes [38].

Future research is warranted to evaluate the metabolic effects of bean consumption in a traditional context among varied groups of human subjects, including those with chronic conditions. The evaluation of additional metabolic outcomes such as inflammatory cytokines, microbial taxa within the gastrointestinal tract and their related metabolites following bean intake would be added strengths. Investigating the knowledge, attitudes, and practices regarding legume consumption among immigrants and native born US individuals is vital for directing dietary guidance from Registered Dietitian Nutritionists and other health providers to ensure they provide meaningful and medically-relevant information to their clients [20].

## 5. Conclusions

Legumes are culturally meaningful foods with numerous health benefits. In the present study, whole black bean and chickpea consumption in combination with white rice, a high glycemic index food, significantly reduced glycemic response in comparison to a white rice control among healthy adult women. Bean treatments were provided in a quantity (1/2 cup) recommended by the 2015 Dietary Guidelines for Americans. Findings have important implications for dietary guidance and retention of traditional food combinations by immigrants, native born US individuals, and others globally.

## Figures and Tables

**Table 1:** Nutrient composition of test meals.

Characteristic	Rice Only Control	Black Beans and White Rice	Chickpeas and White Rice
Total weight (g)	180.0	248.5	248.5
Rice (g)	180.0	118.5	118.5
Beans (g)	---	130.0	130.0
Energy (kcal)	232.0	263.0	258.0
Carbohydrate (g)	49.5	56.1	53.1
Available CHO (g)	49.5	48.6	47.6
Fiber (g)	0.7	7.5	5.5
Protein (g)	4.8	11.2	9.2
Fat (g)	0.5	0.8	2.3

**Table 2:** Descriptive characteristics of women at study entry (*n* = 12).

Characteristic	Mean ± SEM	Range of Values
Age (yrs)	36 ± 4	21–58
Weight (kg)	67.7 ± 2.8	55.9–82.0
Height (cm)	166.8 ± 1.6	160.0–180.3
BMI (kg/m^2^)	23.3 ± 0.9	19.2–28.7
Triglycerides (mg/dL)	100.8 ± 16.6	38–198
Total cholesterol (mg/dL)	180.2 ± 13.6	112–267
LDL (mg/dL)	108.6 ± 13.2	54–183
HDL (mg/dL)	58.2 ± 3.75	41–83
VLDL (mg/dL)	17.9 ± 2.9	7–33
Hemoglobin (g/dL)	14.4 ± 0.5	12.3–16.3
